# Down-regulation of NKD1 increases the invasive potential of non-small-cell lung cancer and correlates with a poor prognosis

**DOI:** 10.1186/1471-2407-11-186

**Published:** 2011-05-20

**Authors:** Sheng Zhang, Yan Wang, Shun-Dong Dai, En-Hua Wang

**Affiliations:** 1 Department of Pathology, the First Affiliated Hospital and College of Basic Medical Sciences of China Medical University, 110001, Shenyang, China

## Abstract

**Background:**

As a negative modulator of the canonical Wnt signaling pathway, Naked1 (NKD1) is widely expressed in many normal tissues. However, the expression pattern and clinicopathological significance of NKD1 in patients with non-small-cell lung cancer (NSCLC) is still unclear.

**Methods:**

Immunohistochemical studies were performed on 35 cases of normal lung tissues and 100 cases of NSCLC, including 66 cases with complete follow-up records. The NKD1 protein and mRNA expressions were detected by western blot and Real-time PCR, respectively. To examine the effect of NKD1 on the invasiveness of lung cancer cells, NKD1 was down-regulated by siRNA in lung cancer cell lines and the invasive ability was then evaluated by the Matrigel invasion assay. In addition, the expressions of Dishevelled-1 and β-catenin proteins, as well as MMP mRNA were also examined in NKD1 knockdown cells.

**Results:**

In 35 fresh lung cancer tissues examined, 27(79%) of them exhibited lower levels of NKD1 protein in comparison with their corresponding normal tissue (P = 0.009). However, the NKD1 mRNA level was significantly higher in cancerous lung tissues, compared with the adjacent normal tissues. In 100 NSCLC tissues, NKD1 was significantly lower in 78 cases (78%) than in the normal specimens, determined by immunohistochemical staining. The reduced NKD1 expression was correlated with histological type (P = 0.003), poor differentiation (P = 0.004), lymph node metastasis (P = 0.013), TNM stage (P = 0.002) and poor survival (62.88 ± 3.23 versus 23.61 ± 2.18 months, P = 0.03). In addition, NKD1 knockdown could up-regulate Dishevelled-1 and β-catenin protein levels, as well as increased MMP-7 transcription and the invasive ability of lung cancer cells. Furthermore, when the NKD1-knockdown cells were treated with Dishevelled-1 antibody, their invasive potential was significantly reduced.

**Conclusion:**

NKD1 protein is reduced but NKD1 mRNA is elevated in NSCLC. Reduced NKD1 protein expression correlates with a poor prognosis in NSCLC. NKD1 might inhibit the activity of the canonical Wnt pathway through Dishevelled-1.

## Background

dNKD (Naked Cuticle Drosophila) was first found in Drosophila and mutation of the Naked gene could induce the loss of segmentation of Drosophila [1]. Subsequently, NKD1 and NKD2, two homologues of drosophila naked cuticle, were also detected in mammalians [2]. These two genes are located on chromosome 16q12.1 and 5p15.3, respectively, in human beings, and share 43.8% amino-acid sequence homology. NKD1 has been proposed to interact with Dishevelled (Dvl) through its conservative domain which forms an EF-hand-like motif, functioning as a negative regulator of the canonical Wnt/β-catenin signaling pathway [3-6]. Dishevelled (Dvl) is a positive regulatory factor located upstream of the Wnt pathway, and plays a role in regulating at least two intracellular Wnt signal pathways, such as the canonical Wnt/β-catenin pathway and JNK/PCP pathway [7].

NKD1 is an antagonist of the canonical Wnt/β-catenin pathway. When it directly interacts with PDZ domain of Dishevelled (Dvl) in the cytoplasm, the canonical Wnt/β-catenin signaling pathway is inhibited. It has been demonstrated that NKD1 could act as a switch that directs Dishevelled (Dvl) toward the JNK/PCP pathway and away from the canonical Wnt/β-catenin pathway, thus inhibiting the canonical Wnt pathway.

Although there are increasing reports of NKD1 today, the role of NKD1 in cancer progression still needs to be further addressed. It has been reported that the NKD1 mRNA level was up-regulated in colorectal adenomas [8] and hepatoblastoma [9]. Conversely, NKD1 protein expression was down-regulated in some gastric cancer tissues. This raises a question of whether the expression level of NKD1 protein is parallel to the mRNA level in the same tumor type. Moreover, to the best of our knowledge, the relationship between NKD1 expression and clinicopathological features in human tumor has not been reported in the English literature.

In the current study, we examine the expression of NKD1 in 100 cases of non-small-cell lung cancer (NSCLC) and correlate it with corresponding clinicopathological factors, including histologic type, neoplastic differentiation, lymph node metastasis and clinical outcome. In addition, we ablated NKD1 by siRNA technology in lung cancer cell lines to investigate alterations in Dishevelled-1 and β-catenin protein levels, MMP-7 transcription and the invasive ability of NKD1-knockdown lung cancer cells, to provide insight into the role of NDK1 in the biological behavior of lung cancers.

## Methods

### Patient and Samples

Paraffin specimens (n = 100) were obtained from patients with lung cancer at the First Affiliated Hospital of China Medical University. Complete follow-up information of 66 patients (30 cases of adenocarcinoma and 36 cases of squamous cell carcinoma) was obtained from review of the patients' medical records. None of these patients had received chemotherapy or radiotherapy before surgical resection. The mean age of these patients was 58 years. According to the WHO histological classification criteria [10], there were 33 cases of squamous cell carcinoma (SCC) and 67 cases of adenocarcinoma (ADC). The p-TNM staging system of the International Union Against Cancer in 2009 [11] was used to classify cases as stages I (n = 24), II (n = 20), III (n = 44), and IV (n = 12).

In addition, 35 paired tissue samples of tumor and parallel non-tumor portion (with >5 cm distance from the primary tumor's edge) for the same case were quickly frozen in liquid nitrogen and stored at -70°C for mRNA and protein analyses.

### Immunohistochemistry

As described previously [12-14], the tissues were fixed with 10% neutral formalin, embedded in paraffin, and 4-μm-thick sections were prepared. All the sections were stained by the streptavidin-peroxidase (S-P) method. Briefly, the sections were incubated with NKD1 polyclonal antibody (1:100, Santa Cruz Biotechnology, Inc.) at 4°C overnight. Meanwhile negative control samples were incubated with non-immune rabbit IgG at the same dilution as for the primary antibody. Biotinylated secondary antibody and diaminobenzidine (DAB) were purchased from Maixin Biotechnology (Fuzhou, China).

### Evaluation of Immunostaining

Five random 200× microscopic fields were examined per slide, and 100 cells were evaluated per field. The expression of NKD1 was classified into five groups according to the percentage of positively staining cells: 0 = absent; 1 = 1-25%; 2 = 26-50%; 3 = 51-75%; 4 = ≥76%. The staining intensity was categorized as follows: 0 = negative; 1 = weak; 2 = moderate; and 3 = strong. The proportion and intensity scores were then multiplied to obtain a total score.

Since the scores in all 35 cases of normal lung tissues were all ≥ 3, we regarded the scores less than 3 as "reduced expression", while scores of 3 or more were considered as "normal expression".

### RT- PCR and Real-Time PCR

Total RNA was extracted with Trizol regent (Invitrogen) according to the manufacturer's instructions. RT-PCR for MMP-7 mRNA was performed with the AMV Ver3.0 kit (Takara, Shiga, Japan). PCR was carried out with the following primers (Table 1). The relative mRNA levels were normalized by the relative amount of β-actin mRNA. Real-Time PCR experimentation for Naked1 mRNA was carried out as follows: Primers were designed and synthesized by Takara Biotechnology (Dalian, China). Reverse transcription of 1 μg RNA was performed with the RT kit (Applied Biosystems, Foster City, CA, USA) and quantitative Real-Time PCR was done using SYBR Green PCR master mix (Applied Biosystems) in a total volume of 20 μL on a 7900HT fast Real-Time PCR system (Applied Biosystems) as follows: 95°C for 30 s, 40 cycles of 95°Cfor 5 s, and 60°C for 30 s. The sequences of the primer pairs were as follows: NKD1 forward, 5^,^-TCGCCGGGATAGAAAACTACA-3^,^, reverse, 5^,^-CAGTTCTGACTTCTGGGCCAC-3^,^; β-actin forward, 5^,^-ATAGCACAGCCTGGATAGCAACGTAC-3^,^, reverse, 5^,^-CACCTTCTACAATGAGCTGCGTGTG-3^,^.

**Table 1:** Primers sequences and reaction condition

		Primer	Length	Temperature	Cycle
**MMP-7**	F:	5'-AGATCCCCCTGCATTTCAGG-3'	163 bp	61°C	35
	R:	5'-TCGAAGTGAGCATCTCCTCC-3'			
**β-Actin**	F:	5'-AGAGCTCAGAGCTGCCTGAC-3'	308 bp	55°C	25
	R:	5'-AGTACTTGCGCTCAGGAGGA-3'			

A dissociation procedure was performed to generate a melting curve for confirmation of amplification specificity. β-actin mRNA was quantified in parallel as the reference control. Relative gene expression was calculated using the Stratagene MxPro software.

### Western Blot

As described previously, total protein was extracted with RIPA lysis buffer and quantified using the Bradford method. Protein lysates (100 μg) were separated by 10% SDS-PAGE and transferred to the polyvinylidene fluoride (PVDF) membranes. After blocking, the blots were incubated with primary antibodies directly against NKD1 Ⅰ(1:200, Santa Cruz), NKD1 Ⅱ (1:500, Cell Signaling) and β-catenin (1:800) at 4°C overnight. After that, the blots were incubated with the secondary antibody labeled with HRP at room temperature for 2 h. Protein bands were visualized using enhanced chemiluminescence (ECL) and detected using the BioImaging System. The relative protein levels were calculated by comparison to the amount of β-actin protein. The experiments were repeated three times independently.

### Cell Culture and Transfection

HBE (the normal human bronchial epithelial cell lines) were obtained from the Cell Bank of Chinese Academy of Science (Shanghai, China). The lung carcinoma cell lines, BE1 and LH7 were kindly provided by Professor Jie Zheng (College of Medicine, Peking University, China). BE1 and LH7 cell lines are two subclones of lung giant cell carcinoma and the BE1 cell line has a high metastatic potential compared with LH7. The β-catenin protein of these two cell lines were both higher than HBE [15]. The Human lung squamous cell line LK and adenocarcinoma cell line LTEP-a-2 (hereafter referred to as LTEP) were purchased from the American Type Culture Collection (Manassas, VA, USA). The cells were cultured in RPMI-1640 (GIBCO, Inc, Grand Island, NY) containing 10% fetal calf serum (Invitrogen), 100 IU ⁄mL penicillin (Sigma, St. Louis, MO, USA), and 100 μg ⁄mL streptomycin (Sigma) in a humidified atmosphere with 5% CO_2_.

NKD1 siRNA (SC-93414) and negative control siRNA (Cat. SC-36869) were all purchased from Santa Cruz Biotechnology (Santa Cruz, CA, USA). For transient transfection, the cells were cultured in a 24-well plate 24 h before the experiment. Then the cells were transfected with Lipofectamine 2000 (Invitrogen, Carlsbad, CA) according to the manufacturer's instructions. Following the transfection, the cells were harvested at 48 hours to measure the mRNA and protein levels.

### Matrigel Invasion Assay

The cells' invasive abilities were examined using a 24-well Transwell with 8-μm pore polycarbonate membrane inserts (Corning Inc., Corning, NY, USA) according to the manufacturer's protocol. Cells that appeared on the lower surface of the filter were counted in five random ×200 fields using an inverted microscope (Olympus IX51; Olympus America Inc, Melville, NY, USA). The experiments were performed in triplicate and got means were calculated.

### Statistical analysis

SPSS version 13.0 for Windows was used for all analyses. The chi-square test was used to examine possible correlations between NKD1 expression and clinicopathological factors. The Student's t -test was used to analyze the results of RT-PCR and western blotting. The Kaplan-Meier method was used for survival analysis, which was measured from the day of surgical resection until death from any cause. The Cox's proportional hazard regression model was used to estimate the possible prognostic significance of clinicopathological variables. P < 0.05 was considered to indicate statistical significance.

## Results

### The protein expression of NKD1 in NSCLC is lower, while mRNA expression is higher than that in normal lung tissue

**Figure 1:**
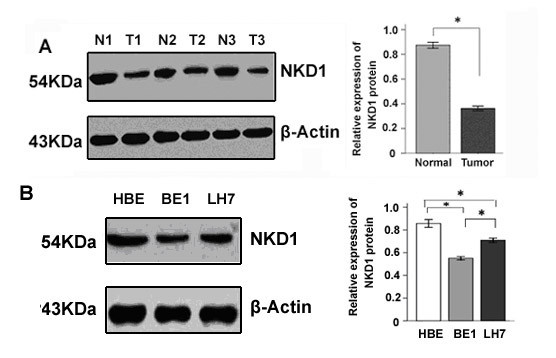
**Expression of NKD1 in lung cancer tissues and cell lines**. (A). Compared with normal lung tissue (N1-N3), NKD1 protein expression was significantly decreased in lung cancer tissues (T1-T3) (P < 0.05). (B). NKD1 protein expression in BE1 and LH7 cells was lower than that in HBE cells, a normal human bronchial epithelial cell line (P < 0.05). In addition, NKD1 protein expression in BE1 cells (high metastatic potential) was lower than that in LH7 cells (low metastatic potential) (P < 0.05).

Total protein of 35 paired NSCLC tissues and adjacent noncancerous lung tissues were extracted and the protein expression of NKD1 was detected by Western blot. The results showed that the expression of NKD1 protein in NSCLC is lower than that in normal lung tissue (P < 0.05, Figure 1A and addition file 1, Figure S1A).

To further examine the expression of NKD1 in cell lines, two lung cancer cell lines (BE1 and LH7) were chosen in our study. HBE, a normal human bronchial epithelial cell line, was used as a control. The results showed that the NKD1 protein expression in HBE was higher than in the lung cancer cell lines. Moreover, NKD1 expression in BE1 cells (high metastatic potential) was lower than that in LH7 cells (low metastatic potential) (Figure 1B and addition file, Figure S1B).

**Figure 2:**
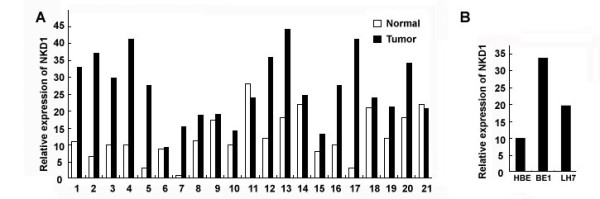
**Real time PCR analyses of NKD1 mRNA in lung cancer tissues and cell lines**. (A). The NKD1 mRNA expression in the lung cancer tissues was significantly higher than in corresponding non-tumorous tissues. (B). NKD1 mRNA expression in BE1 and LH7 cells was higher than that in HBE cells. NKD1 mRNA expression in BE1 cells (high metastatic potential) was higher than in LH7 cells (low metastatic potential).

The above results implied that NKD1 expression might be associated with invasiveness of lung cancer cells. However, Real-time PCR results showed that NKD1 mRNA levels were obviously higher in NSCLC in comparison with corresponding normal lung tissues (Figure 2A). The same results also can be found in cell lines (Figure 2B).

### Reduced NKD1 protein expression was associated with clinicopathological factors and poor prognosis of NSCLC

**Figure 3:**
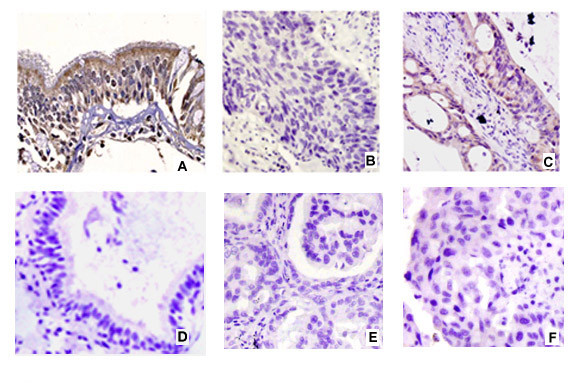
**Immunohistochemical staining of NKD1 in NSCLC**. NKD1 was expressed in the cytoplasm of normal bronchial epithelial cells (Normal expression) (A). NKD1 expression was significantly decreased in lung squamous cell carcinoma (B) and adenocarcinoma (C) (Reduced expression). Negative controls were prepared by non-immune rabbit IgG at the same dilution as for the primary antibody in normal (D) and tumor sample (E and F).

In 35 cases of normal lung tissues examined by immunohistochemistry, NKD1 was mainly expressed in the cytoplasm of bronchial epithelial cells (≥3 score According to our evaluation criteria, they were categorized as normal expression (Figure 3A). In contrast, in the 100 NSCLC specimens, expression of NKD1 was reduced (<3 score) in 78 (78%) samples and was normal (≥3 score) in 22 (22%) samples (Figure 3B and 3C) (Table 2).

**Table 2:** Relationship between NKD1expression and the clinicopathological factors in patients with NSCLC

Clinicopathological factors	n	Normal expression	Reduced expression	**X**^**2**^	P-value
**Age(yr)**					
<58	55	13	42	0.191	0.662
≥58	45	9	36		
**Gender**					
Male	63	15	48	0.325	0.569
Female	37	7	30		
**Histology**					
SCC	33	13	20	8.684	0.003
AC	67	9	58		
**Differentiation**					
Well to moderate	50	17	33	8.392	0.004
Poor	50	5	45		
**pTNM staging**					
I-II	44	16	28	9.447	0.002
III-IV	56	6	50		
**Lymph node metastasis**					
No	45	15	30	6.124	0.013
Yes	55	7	48		

SCC, squamous cell carcinoma; AC, Adenocarcinoma; pTNM staging, pathological TNM staging.

We then analyzed the relationship between NKD1 protein expression and clinicopathological factors in 100 NSCLC samples. The rate of normal NKD1 expression in squamous carcinoma (13/33, 39.4%) was higher than that in adenocarcinoma (9/67, 13.4%) (P = 0.003); the rate of normal NKD1 in stages I-II (16/44, 36.3%) was higher than in stages III-IV (6/56, 10.7%) (P = 0.002); and the rate was also higher in cases without lymphatic metastasis (15/45, 33.3%) than in cases with lymphatic metastasis (7/55, 12.7%) (P = 0.013). In addition, the expression rate of NKD1 in NSCLC with well-moderate differentiation (17/50, 34%) was higher than in the group with poor histologic differentiation (5/50, 10%) (P = 0.004). There was no significant correlation between NKD1 protein expression and gender or age (P > 0.05, Table 2).

**Figure 4:**
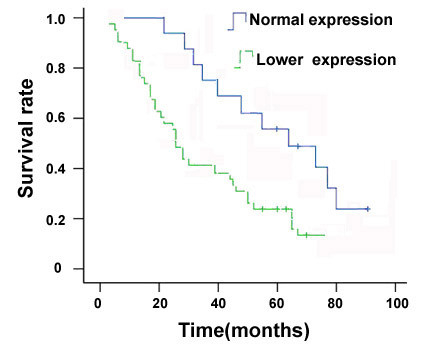
**Relationship between Naked1 expression in NSCLC and postoperative survival period of NSCLC patients**. The Kaplan-Meier curve shows that the overall survival in the patients with lower expression of Naked1 is significantly shorter than in the group with normal expression (p = 0.03).

As shown in Figure 4, Kaplan-Meier survival showed that the mean overall survival in the patients with normal level of NKD1 protein was significantly longer than that in the patients with a low level of NKD1 (62.88 ± 3.23 versus 23.61 ± 2.18 months) ( P = 0.03, Figure 4).

In order to clarify if the reduced NKD1 expression was associated with patients' prognoses, we employed the Cox's proportional hazard regression model, and the result showed that reduced expression of NKD1 protein was a hazard factor for the prognosis of patients with NSCLC (Hazard Ratio = 1.864, with 95% confidence interval = 1.143~2.975; P = 0.017, Table 3).

**Table 3:** Cox regression model for prediction of 100 patients with lung cancer

			95%CI	
Factor	P-value	Hazard Ratio	Lower	Upper
**Age**	0.081	0.612	0.332	1.064
**Gender**	0.276	1.013	0.726	1.998
**Histology**	0.037	0.912	0.665	1.631
**Differentiation**	0.025	0.600	0.354	1.072
**pTNM staging**	0.014	1.554	1.002	2.439
**Lymph node metastasis**	0.002	1.421	1.265	1.726
**Reduced NKD1 expression**	0.017	1.864	1.143	2.975

### NKD1 depletion could up-regulate Dishevelled-1, β-Catenin protein expression and enhance the invasive ability of lung cancer cells

**Figure 5:**
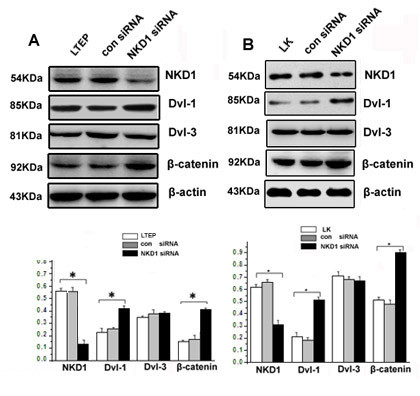
**Knockdown of NKD1 could up-regulate Dishevelled-1 and β-catenin expression in lung cancer cells**. NKD1 knockdown resulted in a significantly reduced expression of NKD1 in LTEP (A) and LK (B) cells. Meanwhile, Dishevelled-1 and β-catenin protein expression was dramatically up-regulated (P < 0.05). Note that Dishevelled-3 expression showed no change in NKD1 knockdown cells (P > 0.05).

After transfection of siRNA-NKD1, the NKD1 protein expression was down-regulated approximately 60% in comparison with control cells (P < 0.05). Correspondingly, the protein expressions of Dishevelled-1 and β-catenin were apparently up-regulated (Figure 5A and 5B).

**Figure 6:**
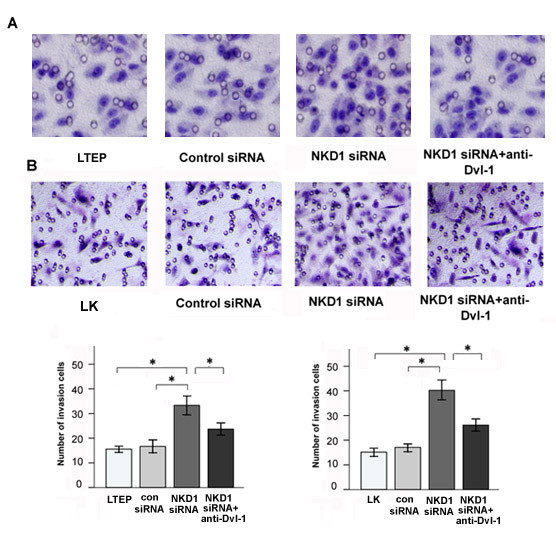
**NKD1 knockdown enhanced the invasive ability of lung cancer cells, while these could be ablated by anti-Dishevelled-1 antibody**. Matrigel invasion assay showed that the normal group had fewer cells invading into the lower surface of the transwell filter than the group transfected with NKD1 siRNA in LTEP (A) and LK (B) lines (P < 0.05). In contrast, When DVL-1 specific antibody incubated with NKD1 siRNA in LTEP (A) and LK (B) lines, the invasive cell number was significantly reduced(P < 0.05).

In order to examine the effect of NKD1 on the invasiveness of lung cancer cells, we down-regulated NKD1 expression in the LTEP and LK cell lines and evaluated the change of their invasive abilities by the Matrigel invasion assay. The results showed that the invasive abilities of the LTEP and LK cells were significantly enhanced when NKD1 was down-regulated by siRNA. When the NKD1-depleted cells were treated with Dvl-1 specific antibody (200 ng/ml), which was used to block Dvl-1 function, the invasive ability of the NKD1-depletion cells was significantly reduced (P < 0.05, Figure 6A and 6B).

**Figure 7:**
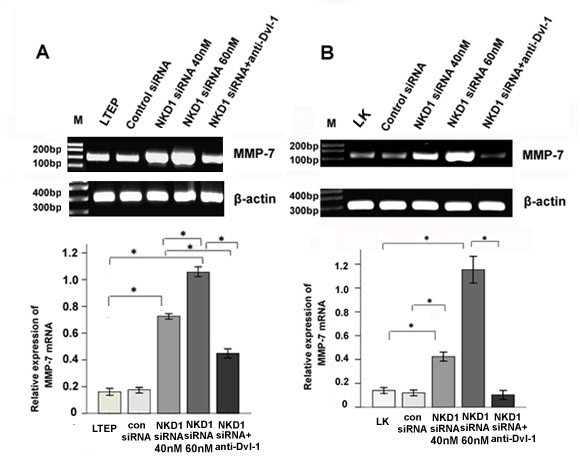
**NKD1 knockdown could enhance the transcriptional activity of MMP-7 gene, and these could be ablated by anti-Dishevelled-1 antibody**. Levels of the MMP-7 gene were gradually increased along with increasing doses of NKD1 siRNA in LTEP (A) and LK (B) cells (P < 0.05). However, when DVL-1 specific antibody incubated with NKD1 siRNA in LTEP (A) and LK (B) lines, MMP-7 mRNA level was decreased significantly (P < 0.05).

These results demonstrated that NKD1 down-regulation could up-regulate Dvl-1, β-catenin protein expression and enhance the invasive ability of lung cancer cells. Since Dvl-1 and β-catenin function as positive regulators of the canonical Wnt signaling pathway, it is plausible that NKD1 may increase the invasive ability of lung cancer cells through activating the canonical Wnt pathway. To test this hypothesis, we examined the expression of MMP-7(a target gene of the canonical Wnt pathway) in NKD1-depleted cells and found that MMP-7 mRNA levels were significantly increased in comparison with lung cancer cells without NKD1 depletion (P < 0.05, Figure 7A and 7B).

## Discussion

NKD1, a negative regulator of the canonical Wnt signaling pathway, is widely expressed in many normal tissues. It has been demonstrated that NKD1 protein was down-regulated in gastric cancer, whereas its mRNA expression was up-regulated in colorectal adenomas and hepatoblastoma. However, it is currently unclear whether or not NKD1 protein expression is in parallel to the mRNA expression in the same individual tumor. In addition, the relationship between NKD1 and clinicopathological factors needs to be further clarified. In order to provide insight into these clinicopathological aspects, we used paired NSCLC/normal tissues and lung cancer cell lines to investigate the NKD1 expression at both mRNA and protein levels, and correlated the data from NSCLC tissues with pathologic and clinical findings in the individual patients.

The NKD1 protein expression in NSCLC tissues was lower than that in normal lung tissue in our study. Theoretically, this would result in activating the canonical Wnt pathway by losing NKD1's negative regulatory effect in the pathway. Our immunohistochemical results demonstrated that reduced NKD1 expression was associated with poor differentiation, high pTNM stage, lymph node metastasis and poor prognosis in NSCLC. This interpretation is in keeping with the results obtained from our in vitro experiments on BE1 and LH7 cells, which were two sub-clones derived from a single lung cancer cell line. The BE1 cells which have demonstrated high metastatic potential showed much lower levels of NKD1 than the LH7 cells that have low metastasis. Furthermore, our NKD1 knockdown assays using the siRNA technology revealed an enhanced invasive ability in LTEP and LK cells, supporting the notion that loss of NKD1 activity might critically contribute to the metastatic potential in lung cancer cells.

In order to explore how NKD1 knockdown increases the invasive ability of lung cancer cells, we measured the protein expressions of Dvl-1, Dvl-3 and β-catenin, the key protein factors involved in the Wnt pathway, as well as the transcriptional expression of MMP-7, the down-stream gene of Wnt pathway [16-18], in NKD1 down-regulated cells. The results showed that NKD1 knockdown caused dramatically up-regulated protein expression of two positive modulators of the canonical Wnt pathway, Dvl-1 and β-catenin as well as enhanced transcription of the MMP-7 gene. These data suggested that NKD1 knockdown could activated the Wnt pathway in LTEP and LK cell lines. Recently, Yan D *et al *demonstrated that NKD1 could up-regulate β-catenin protein expression in human colon tumors, and Hu T *et al *reported NKD2, a homologue of NKD1, could accelerate the degradation of Dishevelled by increasing its polyubiquitylation [19]. Although our data are similar to those being reported, whether the same mechanism is involved in modulation of Dishevelled by NKD1 and NKD2 remains to be further studied.

Using a loss-of-function approach with a Dishevelled-1 specific antibody, NKD1 knockdown LTEP and LK cells were shown to have an increased transcription of MMP-7 and an enhanced invasive ability. The result further indicated that NKD1 might play a role as a negative regulator of the canonical Wnt signaling pathway in these two lung cancer cell lines. Moreover, we found that NKD1 knockdown markedly up-regulated Dvl-1 protein expression but did not significantly change Dvl-3 protein expression. We have previously demonstrated that Dvl-1 mainly involved the canonical Wnt pathway, while Dvl-3 primarily regulated the activity of non-canonical Wnt pathway in lung cancer cells [20]; thereby, expression of MMP-7 was chosen as a marker to reflect the activity of the classical Wnt signal pathway in our study.

Interestingly, the NKD1 protein expression in NSCLC is lower than in normal lung tissue, whereas the mRNA expression in NSCLC is higher than in normal lung tissue. These data were consistent with the previous reports, and demonstrated that NKD1 protein expression was dissociated with its mRNA expression at least in NSCLC. We speculate the enhanced NKD1 mRNA level might be due to the elevated activity of Wnt pathway in NSCLC, given that the NKD1 gene is one of the target genes down-stream of the classical Wnt pathway. This is a probable feed-back mechanism. In NSCLC, this feed-back loop is formed by a decrease inNKD1 protein → increase in β-catenin protein → activation of Wnt pathway→up-regulation of NKD1 mRNA transcription. However, this feed-back loop seems to be incomplete, given the enigmatic low level of NKD1 protein despite a high level of mRNA. Previous study on both colorectal carcinomas and hepatoblastomas has suggested that up-regulation of NKD1 mRNA could be a marker of activation of the Wnt signaling pathway in those tumors; while this up-regulation seemed to correlate with β-catenin mutational status in colon cancer, it was generalized in hepatoblastomas. Moreover, we have previously demonstrated that there was no β-catenin mutation in NSCLC [21], and when NKD1 was knocked down in LK and LTEP cell lines, β-catenin protein was up-regulated. This suggested that the up-regulation of β-catenin protein in NSCLC was not due to the mutation of β-catenin, but may be related to the down regulation of NKD1 protein. However, the detailed mechanism in which NKD1 regulates the expression of β-catenin in NSCLC remains to be further investigated.

How NKD1 protein expression was reduced in NSCLC with up-regulated NKD1 mRNA is an essential question to be clarified. The mechanisms may involve a post-translation modification of NKD1, and/or the process of protein degradation. Theoretically, as a negative-feedback loop exists to control the level of NKD1 protein, the high expression of NKD1 protein might inhibit the classical Wnt signal pathway and thereby inhibit the transcription of NKD1 [22]. Conversely, reduced NKD1 protein might enhance the activity of the classical Wnt pathway and up-regulate the transcription and translation of NKD1 gene, thus promoting the NKD1 protein expression. However, this final step of the feed-back balancing mechanism might be disrupted in cancer cells. The NKD1 protein degradation might be accelerated or the protein might be more fragile due to the altered post-translation modifications of the protein. Thereby, the NKD1 protein tends to beat a low level in lung cancer cells, even though the classical Wnt pathway is constantly shown to be activated. Certainly, more experimental studies are needed to test our hypothesis.

## Conclusion

We found that NKD1 protein was down-regulated in lung cancer tissues, which was associated with poor differentiation, high pTNM stage, lymph node metastasis and poor prognosis of NSCLC. Interestingly, NKD1 protein in NSCLC was lower than that in normal lung tissue, whereas the mRNA expression in NSCLC is higher than that in normal lung tissue. In addition, NKD1 knockdown by siRNA technology could markedly up-regulate Dishevelled-1 and β-catenin protein levels, enhance the transcription of MMP-7 and promote the invasiveness of lung cancer cells. Furthermore, when the NKD1-knockdown cells were treated with Dishevelled-1 antibody, their invasive ability was significantly reduced. Our data support the nation that the decreased NKD1 protein could relieve its inhibitory effect on the Wnt pathway, and activate it by up-regulating Dishevelled-1 in NSCLC.

## Abbreviations

NKD1: Naked1; Dvl: Dishevelled; NSCLC: non-small cell lung cancer; MMP-7: matrix metallopeptidase 7.

## Competing interests

The authors declare that they have no competing interests.

## Authors' contributions

SZ carried out prepared pathology samples, performed immunohistochemical studies, cell culture studies, invasive assays and wrote the manuscript. EHW designed research, evaluated immunohistochemical results, and wrote/revised the manuscript. SDD and YW involved in the statistical analyses of all data. The manuscript has been read and approved by all the authors.

## Pre-publication history

The pre-publication history for this paper can be accessed here:

http://www.biomedcentral.com/1471-2407/11/186/prepub

## Supplementary Material

Additional file 1 **Figure S1. The expression of NKD1 protein in lung tissues and cell lines with NKD1 antibody II**. (A). Compared with normal lung tissue (N1-N3), NKD1 protein expression was significantly decreased in lung cancer tissues (T1-T3) (P < 0.05). (B). NKD1 protein expression in BE1 and LH7 cells was significantly lower than that in HBE cells, a normal human bronchial epithelial cell line (P < 0.05). Click here for file
