# Supplementary figures and images for: Down-regulation of NKD1 increases the invasive potential of non-small-cell lung cancer and correlates with a poor prognosis
Source: BMC Cancer. 2011 May 20;11:186. doi: 10.1186/1471-2407-11-186 (PMC3118196; doi:10.1186/1471-2407-11-186)

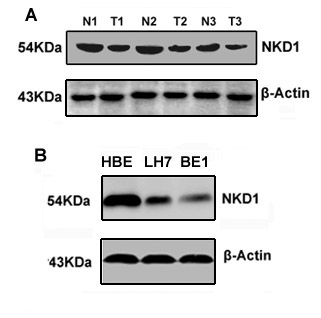

Supplement: Additional file 1 — Figure S1. The expression of NKD1 protein in lung tissues and cell lines with NKD1 antibody II. (A). Compared with normal lung tissue (N1-N3), NKD1 protein expression was significantly decreased in lung cancer tissues (T1-T3) (P < 0.05). (B). NKD1 protein expression in BE1 and LH7 cells was significantly lower than that in HBE cells, a normal human bronchial epithelial cell line (P < 0.05). [file 1471-2407-11-186-S1.JPEG]
